# Quorum Sensing and Density-Dependent Dispersal in an Aquatic Model System

**DOI:** 10.1371/journal.pone.0048436

**Published:** 2012-11-07

**Authors:** Simon Fellous, Alison Duncan, Aurélie Coulon, Oliver Kaltz

**Affiliations:** 1 Institut des Sciences de l’Evolution UMR 5554, Université Montpellier 2– CNRS, Montpellier, France; 2 Centre de Biologie pour la Gestion des Populations, INRA, Montferrier sur Lez, France; 3 Conservation des Espèces, Restauration et Suivi des Populations UMR 7204, Muséum National d’Histoire Naturelle, Brunoy, France; CNRS, Université de Bourgogne, France

## Abstract

Many organisms use cues to decide whether to disperse or not, especially those related to the composition of their environment. Dispersal hence sometimes depends on population density, which can be important for the dynamics and evolution of sub-divided populations. But very little is known about the factors that organisms use to inform their dispersal decision. We investigated the cues underlying density-dependent dispersal in inter-connected microcosms of the freshwater protozoan *Paramecium caudatum*. In two experiments, we manipulated (i) the number of cells per microcosm and (ii) the origin of their culture medium (supernatant from high- or low-density populations). We found a negative relationship between population density and rates of dispersal, suggesting the use of physical cues. There was no significant effect of culture medium origin on dispersal and thus no support for chemical cues usage. These results suggest that the perception of density – and as a result, the decision to disperse – in this organism can be based on physical factors. This type of quorum sensing may be an adaptation optimizing small scale monitoring of the environment and swarm formation in open water.

## Introduction

Dispersal influences the population dynamics and genetic distribution of organisms within spatially structured populations. In animal systems, dispersal is often the result of a choice by the organism to either stay in its current patch or to move to another patch where it might reproduce [1]. Numerous biotic and abiotic factors are known to influence the different aspects of dispersal behavior (propensity to disperse, dispersal direction, distance and trajectory, destination). Environmental quality, hormonal status, parasitic state and genetic properties are but a few examples [2].

Demographical states such as population density can also influence this behaviour. Positive density-dependence in dispersal (where emigration propensity increases with increasing population density) has been shown in a variety of taxa [3], [4]. It can be explained by the increased competition for resources and abiotic stress associated with higher densities [3], [5]. These negative effects associated with high density are intuitively logical and therefore theoretical models commonly assume positive density-dependent dispersal [5]. Modeling the evolution of density-dependent dispersal shows that depending on environmental conditions the strength of the positive relationship (i.e. the slope) between density and dispersal can greatly vary [6], [7]. However, high conspecific density can also be advantageous, e.g., for social foraging, assessment of resource availability, or the avoidance of an Allee effect. Consequently, dispersal may become negatively linked to population density, as has been observed in several cases [3], [5], [8]. Because of the challenges associated with manipulating population size in free-living organisms, most studies linking dispersal to density show a correlation without investigating the causal mechanisms (but see e.g. [9]). Consequently it is unclear whether the correlation is due to another external factor acting simultaneously on population density and dispersal behavior. Here we use microcosms of the aquatic protozoan *Paramecium caudatum* to investigate the factors driving the dispersal behavior of this organism.

We recently observed negative density-dependent dispersal in an experiment with the ciliate *P. caudatum*: clonal populations with higher densities had the lowest levels of dispersal between experimental microcosms [10]. However, in this study population densities were not experimentally manipulated, but were emerging properties of the populations, which also varied in genotype, infection status and microenvironment. Low dispersal of high density populations may thus have been triggered by another, unidentified factor. The present study aimed at (i) experimentally testing whether population density really governs dispersal of *P. caudatum* and (ii) investigating possible cues by which this ciliate perceives population density (i.e. quorum sensing) and decides to disperse.

We manipulated population density or culture medium in two microcosm experiments and tested whether density-dependent dispersal was triggered by chemical or physical cues. Ciliates in general, and *Paramecium* in particular, are known to respond to both types of cues by changes in swimming behaviour [11], [12] and therefore possibly dispersal. In fact, Hauzy et al. (2007) have demonstrated the use of chemical factors as cues for dispersal in two ciliates. Not only did *Dileptus* predators disperse less at high densities of their *Tetrahymena* prey, but they also responded to filtered prey medium, indicating a chemical cue [4]. Ogata et al (2008) report reduced swimming speed in aggregated clusters of *P. multinucleatum*, suggesting a link between density and dispersal [11]. Paramecia may use molecules released by conspecifics to estimate population density and to inform their dispersal decisions. To test for this possibility, we measured dispersal rates of *P. caudatum* populations exposed to medium from high- or low-density cultures.

Alternatively, direct physical contact may serve as a cue for population density. Physical contact with objects causes membrane depolarization and a subsequent movement response by the *Paramecium* (attraction, avoidance; [12]). Changes in direction and speed of swimming can also be seen when individual paramecia bump into each other in laboratory assemblages [13]. Thus variation in population density may translate into variation in cell encounter rate and thereby influence swimming behaviour and dispersal propensity. We tested this hypothesis by manipulating the density of *P. caudatum* populations, while keeping their medium unchanged.

## Materials and Methods

### 1. Biological System

The protozoan *P. caudatum* inhabits stagnant freshwater bodies of the Northern Hemisphere [12]. The same mitochondrial haplotypes can be found thousands of kilometres apart, but also coexist in the same pond [14]. Our laboratory cultures are maintained at 23°C, in a medium prepared from dried organic lettuce supplemented with the food bacterium *Serratia marcescens*.

We used 6 genetically distinct clones originating from Poland (CRA), Japan (K8), Germany (TUB, M3 and GÖR) and Italy (VEN). Populations of each of these clones were cultured in 50 ml plastic tubes. Large portions of their media were regularly changed in the weeks prior to the experiment so that all populations were in similar demographic states at the beginning of the experiment (i.e. regularly dividing and with densities close to carrying capacity). Density at carrying capacity differed among clones (range: c. 70–600 cells per ml).

### 2. General Experimental Set-up

We assayed the dispersal behaviour of *P. caudatum* using a device for ciliate dispersal studies established by [15] and also used by Fellous et al. (2011) (see for similar devices: [4], [16]). Briefly, we used mazes made of three 5-ml plastic tubes, connected by 5 cm-long rubber tubing (diameter 3 mm). Connections between these tubes can be closed with clamps (Fig. S1). In each experimental unit, a known number of *P. caudatum* cells and 3.25 mL of medium were placed in the central tube, while fresh medium was added to the two lateral tubes, (each tube containing 3.25 ml of liquid during the assay). After paramecium introduction, the clamps were removed, allowing the paramecia to move freely between tubes. After 3 h, the number of paramecia in the two lateral tubes (dispersers) were counted. For this short dispersal period, total population size remains constant [10] and therefore mortality and growth are unlikely to affect dispersal. Treatment order was randomized and tube identity unknown to the experimenter. Manipulation of population densities and medium was done by centrifuging populations for 10 minutes at 2600 g and separating the supernatant from the paramecium-containing pellet. We then mixed supernatant and pellets in different ratios to create our experimental treatments.

### 3. Experiment 1

In experiment 1, we manipulated the medium in which the cells were assayed, but not the population density. The rationale of this treatment was to test for chemical cues in the medium related to population density. To this end, we assayed dispersal of paramecia exposed to donor medium from populations with either higher or lower density; paramecia were also exposed to their own medium.

We used paramecia from the 6 clones described above, with natural densities ranging from 76 to 585 cells per ml; each clone was represented by two replicate cultures of very similar densities. After centrifugation, different combinations of pellets (containing the paramecia) and donor medium (i.e., supernatant) were established. Thus replicate populations of each clone were tested in their own medium type (replicated twice) and in the medium from 2–3 other ‘donor’ clones (each replicated once), giving a total of 26 replicates. After 3 h, we estimated dispersal rate from counts of the number of dispersers in the lateral tubes and the number of paramecia remaining in the central tube.

### 4. Experiment 2

In experiment 2, we manipulated population density, but kept the paramecia in their own medium. By adding together appropriate amounts of paramecia from centrifuged pellets and supernatant, we adjusted population density to three levels: 250, 750 or 1500 individuals per tube (i.e. 77, 230 and 460 cells per ml). Paramecia from two independent replicate populations were tested for each of three clones (Cra, K8 and Grö). The two lower densities were established for all replicate populations; the highest density could only be established for the two replicate populations of the K8 clone (this clone had the highest natural density with c. 400 individuals per ml; clone Cra and Grö harbored 80 and 170 individuals per ml respectively). This gave a total of 14 independent assays (n = 3 clones×2 tubes of origin×2 or 3 treatments).

### 5. Statistical Analyses

We used general linear models for data analysis. The response variable was the proportion of paramecia that dispersed within a given experimental apparatus (its distribution complied with linear model assumptions).

In experiment 1, the statistical model contained two continuous explanatory variables: the natural density of the recipient population and the density of the population from which the donor medium was taken. We further added clone identity of the recipient population and clone identity of the donor population to the model. Clone identity was treated as a fixed factor since the clones were deliberately chosen for their different natural densities. Because density and clone were partly confounded, they were each tested sequentially (type 1 tests): the p-values that we report for each of these two terms are those when they are entered into the model first. A power analysis was also carried out on the effect of density in the donor population.

For analysis of experiment 2, we fitted clone identity, replicate population (nested within clone) and experimentally manipulated population density to the model.

After fitting all relevant terms (including 2-way interactions) we progressively removed the non-significant ones (backward model selection). The results presented in Table 1 are terms from final models when significant, as well as tests of the factors of interest (when added one by one to the final model). Analyses were carried with the JMP 8 statistical package.

**Table 1:** Statistical analysis of the proportion of paramecium cells dispersing in experiments 1 and 2.

	Factor	F	d.f.	P
Experiment 1: Manipulation ofculture medium	Clone identity^1^	2.98	5, 17	0.041
	Log (population density in the experimental apparatus)^1^	.29	1, 17	0.023
	Log (population density previously contained by thedonor medium)	0.29	1, 21	0.60
	Clone identity of the donor medium population	1.67	5, 17	0.19
	Donor and assayed paramecia are from the sameor a different clone.	0.01	1, 21	0.92
Experiment 2: Manipulation ofpopulation density	Clone	14.1	2, 7	0.003
	Log (experimental population density)	12.6	1, 7	0.009
	Tube of origin [nested in Clone]	5.99	3, 7	0.024

The values provided for significant terms are those in the final model; for the others, the values are those when added one by one to the final model.

1 because clone and initial density are partly confounded, these two terms were not significant when added simultaneously to the model (SAS-type 3 fitting), but each was significant when added first in sequential (type 1) fitting procedure.

## Results

### 1. Experiment 1: Test for Chemical Signaling

**Figure 1:**
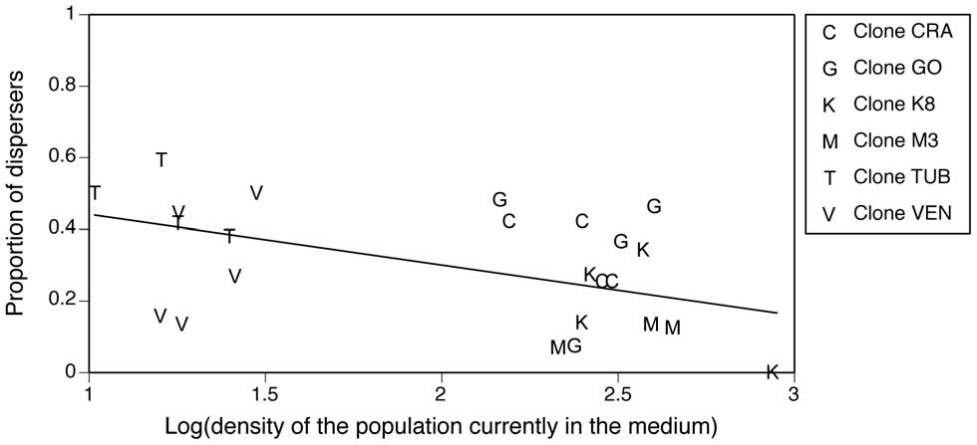
Relationship between population density and the proportion of dispersing cells in experiment 1. As in Fellous et al. (2011) we find a significant negative correlation between the natural concentration of paramecium cells and dispersal. Each point represents an independent replicate.

**Figure 2:**
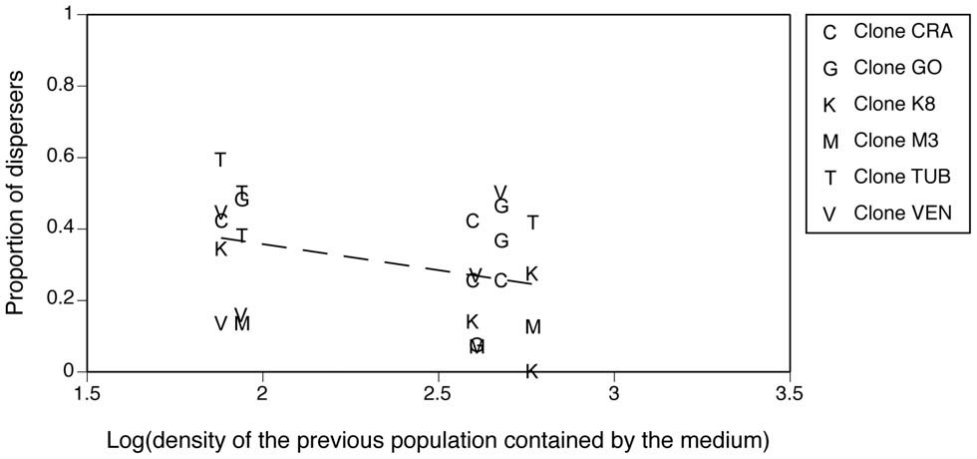
Relationship between the donor population density (i.e. density of the population previously contained by the culture medium) and the proportion of dispersing cells (experiment 1). In this experiment, we manipulated the nature of the medium but not the cell concentration. Populations were exposed to medium from donor populations of higher or lower density. The non-significant relationship does not support the chemical mediation hypothesis. Each point represents an independent replicate.

The proportion of dispersing cells decreased with increasing population density (Table 1, Fig. 1), thus exhibiting negative density-dependent dispersal. However, differences in population density were partly confounded with *Paramecium* clone identity: sequential (type 1) test confirmed the significant effects of clone identity and population density in the experimental apparatus (Table 1). Dispersal was not significantly affected by the donor medium, in which the cells were assayed: neither the density of the donor population, nor donor clone identity was significant (Table 1; Fig. 2). There also was no significant effect of testing paramecia in a medium that had previously contained their clone or a different one (Table 1). A power analysis on donor medium density revealed a 0.136 probability of mistakenly concluding an absence of effect of this factor.

### 2. Experiment 2: Test of Physical Interactions

**Figure 3:**
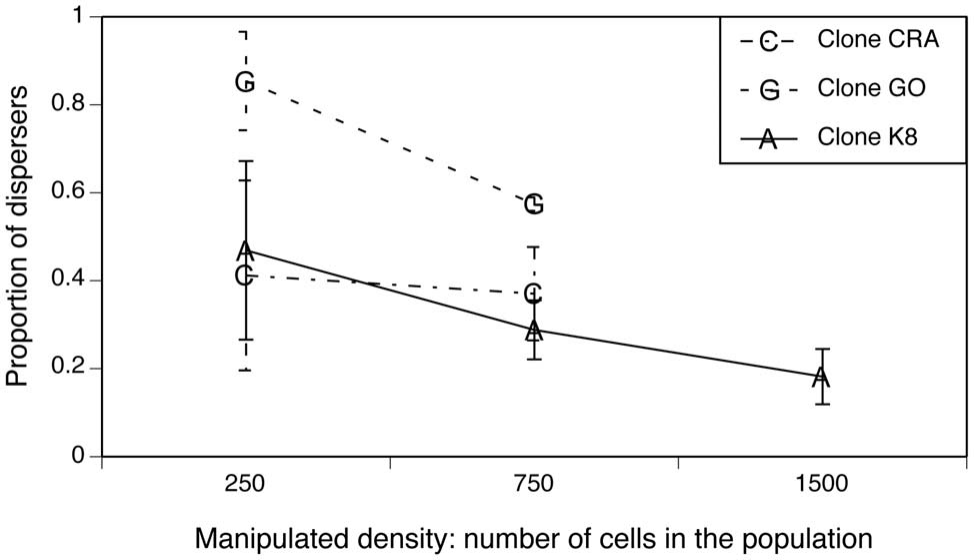
Relationship between experimental population density and the proportion of dispersing cells (experiment 2). We manipulated paramecium cell density, but not their medium. The significant relationship between cell density and dispersal supports the hypothesis that paramecium use physical interactions as cues regarding population density. Symbols indicate means and error bars represent standard errors.

The proportion of dispersing cells decreased when population density increased (Fig. 3, Table 1), again demonstrating negative density-dependent dispersal. At the lowest density (250 paramecia per population), nearly 60% of cells had dispersed after 3 hours, while only 40% dispersed from populations set to 750 individuals; at a density of 1500 individuals (K8 clone only), dispersal dropped to 18%. Paramecium clone identity was a significant determinant of dispersal (Fig. 3, Table 1).

## Discussion

In our experiments, dispersal of the ciliate *Paramecium caudatum* was negatively linked to population density. An artificial increase in the number of cells in the medium led to reduced dispersal, while manipulating the cells’ medium had no significant effect on their dispersal behaviour. These results do not support the idea that dispersal is triggered by density-related concentrations of chemical cues in the environment. Instead, the results are consistent with the hypothesis that the paramecia use physical interactions for quorum sensing and thus inform their dispersal decision.

Our finding of negative density-dependent dispersal corroborates results from a recent study by Fellous et al (2011). This tendency to remain grouped when at high density is also consistent with certain quasi-social features of this organism. It is well known that paramecia aggregate and show coordinated unidirectional swimming behaviour in the laboratory [11]. Similarly, in its natural habitat, stagnant water bodies, *P. caudatum* has a very heterogeneous, patchy spatial distribution [17]. Thus, negative density-dependent dispersal, as we report here, could be one mechanism to reinforce aggregation and swarming.

Evolutionary theory suggests a number of possible causes of negative density-dependent dispersal. Some of them are based on kinship, avoidance of Allee effects or optimal habitat exploitation [3]. In *P. caudatum*, Allee effects (i.e., the reduced growth of low density populations) may exist. At least in the laboratory, it is sometimes observed that populations comprising a few individuals are more likely to go extinct or show lower growth than larger populations. The mechanism for this apparent Allee effect is unknown.

Alternatively, lacking a complex neural system, paramecia may use population density as an indicator of environmental quality. The presence of many conspecifics may indicate good patch quality, e.g. high food supply or absence of predators. This may act as an incentive to remain in dense aggregations, thereby causing a negative relationship between density and dispersal. This form of quorum sensing may efficiently integrate and summarise information collected by many individuals [18]. Indeed, “informed dispersal” has been demonstrated in common lizards [18], whereby they learn about the density of surrounding populations through some (unknown) traits of immigrants; they use this piece of social information in their decision to disperse [19], [20]. Recent theory however shows that informed dispersal may not always be optimal, in particular when the environment is unpredictable and the acquisition of information costly [21]. In the case of clonal organisms, such as *P. caudatum*, a bet-edging strategy may emerge with a fraction of the population dispersing. Environmental assessment mechanisms, and their inherent costs, would thus be key to the evolution of context-dependent dispersal.

Presumably, lizards have better cognitive abilities than paramecia. However, even a unicellular ciliate may be capable of collecting and treating this type of information. We had two *a priori* hypotheses regarding the nature of the cue used by *P. caudatum* for quorum sensing – namely the sensing of excreted molecules and the use of physical information. Our experimental manipulation of culture medium provided no evidence for chemical cues, but *Paramecium* dispersal seemed to respond to the physical presence of conspecifics. The nature of this physical factor remains unidentified. Other ciliate species detect the presence of predator by direct membrane contact or use the hydrodynamic disturbances induced by cilia motion [22], [23]. Direct contact between individual *Paramecium* cells can change swimming speed and direction [13], and the frequency of these contacts likely correlates with population density. We thus hypothesise that *P. caudatum* reduces its dispersal according to how often it encounters direct physical contact with a conspecific. This hypothesis is also consistent with a negative correlation between *Paramecium* density and individual swimming speed observed by [11], although these authors assume this effect to be mediated by chemical attraction.

We are nonetheless cautious about this hypothesis, as other mechanisms could be involved. First, the power analysis revealed a 0.13 probability that paramecia used chemical cues even though the test was not significant at the α = 0.05 level. Indeed, the relationship between density in the donor medium and dispersal is negative, as expected under the chemical signaling hypothesis. Second, we cannot rule out the action of short-lived chemical factors, which may degrade too rapidly to induce an effect in our second experiment (but see second to last paragraph for a discussion of *Paramecium* use of chemicals in water). Third, it is also possible that other types of physical factors are involved. For example, Fels (2008) proposed that emission of low intensity light (i.e., biophotons) may regulate population growth in *Paramecium* [24]. Finally, when centrifuging the paramecia for the density manipulation experiment, we may not only have concentrated the paramecia, but also the food bacteria present in the culture. Therefore, treatments with higher paramecium densities could also have contained more bacteria, possibly inciting *Paramecium* to stay where food is abundant. However, in an additional experiment, naturally high paramecia densities were not associated with high bacterial numbers (unlike low dispersal levels) allowing us to rule out this possibility (see Fig. S2 and Text S1).

The best cue for monitoring population status will depend on the environment in which an organism lives. It was recently shown that the worm *Caenorhabditis elegans* exhibits positive density-dependent dispersal and uses odors to decide whether to disperse or not [25]. These worms live in the soil, an environment with a strong spatial structure [26] restricting molecule diffusion. By contrast, paramecia live in open water-bodies in which emitted molecules can easily be diluted and carry information on long distances. Using chemical cues to monitor population density may therefore be difficult, in particular at a small spatial scale. Hence the use of physical factors such as encounter rates may be a more reliable alternative and permit swarm formation.

Our results demonstrate the causative influence of crowding on dispersal. This observation contrasts with the majority of studies where population density and dispersal propensity are correlated without excluding the possibility that other extrinsic factors influence both parameters [2], [10]. Density-dependent dispersal is generally assumed to be positive, but negative relationships such as we show here may be frequent and have important consequences for meta- and sub-population dynamics [3]. Finally, our experiments shed light on the mechanisms behind *P. caudatum*’s negative density-dependence dispersal and discuss how the specific environmental cues employed are linked to an organism’s lifestyle and habitat.

## Supporting Information

Figure S1 (TIFF)

Figure S2 (TIFF)

Text S1 (DOC)

## References

[1] Ronce O (2007) How Does It Feel to Be Like a Rolling Stone? Ten Questions About Dispersal Evolution. Annual Review of Ecology and Systematics 38: 231–253.

[2] Clobert J, Anker R, Rousset F (2004) Causes, mechanisms ad consequences of dispersal. In: Hanski I, Gaggiotti O, editors. Ecology, Genetics and Evolution of metpopulations. Burlington: Elsevier Academic Press. 307–336.

[3] Bowler DE, Benton TG (2005) Causes and consequences of animal dispersal strategies: relating individual behaviour to spatial dynamics. Biological Reviews 80: 205–225. 15921049 10.1017/s1464793104006645

[4] Hauzy C, Hulot FD, Gins A, Loreau M (2007) Intra- and interspecific density-dependent dispersal in an aquatic prey-predator system. The Journal of animal ecology 76: 552–558. 17439471 10.1111/j.1365-2656.2007.01227.x

[5] Matthysen E (2005) Density-dependent dispersal in birds and mammals. Ecography 28: 403–416.

[6] Travis JMJ, Mustin K, Benton TG, Dytham C (2009) Accelerating invasion rates result from the evolution of density-dependent dispersal. Journal of Theoretical Biology 259: 151–158. 19289134 10.1016/j.jtbi.2009.03.008

[7] Poethke HJ, Hovestadt T (2002) Evolution of density- and patch-size-dependent dispersal rates. Proceedings of the Royal Society of London Series B: Biological Sciences 269: 637–645. 11916481 10.1098/rspb.2001.1936 PMC1690934

[8] Kim SY, Torres R, Drummond H (2009) Simultaneous positive and negative density-dependent dispersal in a colonial bird species. Ecology 90: 230–239. 19294928 10.1890/08-0133.1

[9] Le Galliard JF, Ferrière R, Clobert J (2003) Mother-offspring interactions affect natal dispersal in a lizard. Proceedings of the Royal Society of London Series B: Biological Sciences 270: 1163–1169. 12816655 10.1098/rspb.2003.2360 PMC1691359

[10] Fellous S, Quillery E, Duncan AB, Kaltz O (2011) Parasitic infection reduces dispersal of ciliate host. Biology Letters 7: 327–329. 20961885 10.1098/rsbl.2010.0862 PMC3097846

[11] Ogata M, Hondou T, Hayakawa Y, Hayashi Y, Sugawara K (2008) Adaptation-induced collective dynamics of a single-cell protozoan. Physical Review E 77: 011917. 10.1103/PhysRevE.77.011917 18351886

[12] Wichterman R (1986) The biology of *Paramecium*. New York City: Plenum Press.

[13] Ishikawa T, Hota M (2006) Interaction of two swimming Paramecia. Journal of Experimental Biology 209: 4452–4463. 17079716 10.1242/jeb.02537

[14] Barth D, Krenek S, Fokin SI, Berendonk TU (2006) Intraspecific genetic variation in *Paramecium* revealed by mitochondrial cytochrome C oxidase I sequences. Journal of Eukaryotic Microbiology 53: 20–25. 16441579 10.1111/j.1550-7408.2005.00068.x

[15] Fjerdingstad E, Schtickzelle N, Manhes P, Gutierrez A, Clobert J (2007) Evolution of dispersal and life history strategies - *Tetrahymena* ciliates. BMC Evolutionary Biology 7: 133. 17683620 10.1186/1471-2148-7-133 PMC1997130

[16] Altermatt F, Holyoak M (2012) Spatial clustering of habitat structure effects patterns of community composition and diversity. Ecology In press. 10.1890/11-1190.1 22764498

[17] Görtz HD (1988) *Paramecium*. Berlin: Springer-Verlag.

[18] Clobert J, Le Galliard JF, Cote J, Meylan S, Massot M (2009) Informed dispersal, heterogeneity in animal dispersal syndromes and the dynamics of spatially structured populations. Ecology letters 12: 197–209. 19170731 10.1111/j.1461-0248.2008.01267.x

[19] Cote J, Boudsocq S, Clobert J (2008) Density, social information, and space use in the common lizard (*Lacerta vivipara*). Behavioral Ecology 19: 163–168.

[20] Cote J, Clobert J (2007) Social information and emigration: lessons from immigrants. Ecology letters 10: 411–417. 17498140 10.1111/j.1461-0248.2007.01032.x

[21] Bocedi G, Heinonen J, Travis J (2012) Uncertainty and the role of information acquisition in the evolution of context-dependent emigration. The American naturalist 179: 606. 10.1086/665004 22504543

[22] Kuhlmann H (1994) Escape response of *Euplotes octocarinatus* to turbellarian predators. Archives of Protistenkd 144: 163–171.

[23] Kusch J (1993) Behavioural and morphological changes in ciliates induced by the predator *Amoeba proteus* . Oecologia 96: 354–359. 28313650 10.1007/BF00317505

[24] Fels D (2009) Cellular Communication through Light. PLoS ONE 4: e5086. 19340303 10.1371/journal.pone.0005086 PMC2660427

[25] Yamada K, Hirotsu T, Matsuki M, Butcher RA, Tomioka M, et al (2010) Olfactory Plasticity Is Regulated by Pheromonal Signaling in *Caenorhabditis elegans* . Science 329: 1647–1650. 20929849 10.1126/science.1192020 PMC3021133

[26] Vos M, Birkett PJ, Birch E, Griffiths RI, Buckling A (2009) Local Adaptation of Bacteriophages to Their Bacterial Hosts in Soil. Science 325: 833–833. 19679806 10.1126/science.1174173

