# Supplementary figures and images for: Quorum Sensing and Density-Dependent Dispersal in an Aquatic Model System
Source: PLoS One. 2012 Nov 7;7(11):e48436. doi: 10.1371/journal.pone.0048436 (PMC3492347; doi:10.1371/journal.pone.0048436)

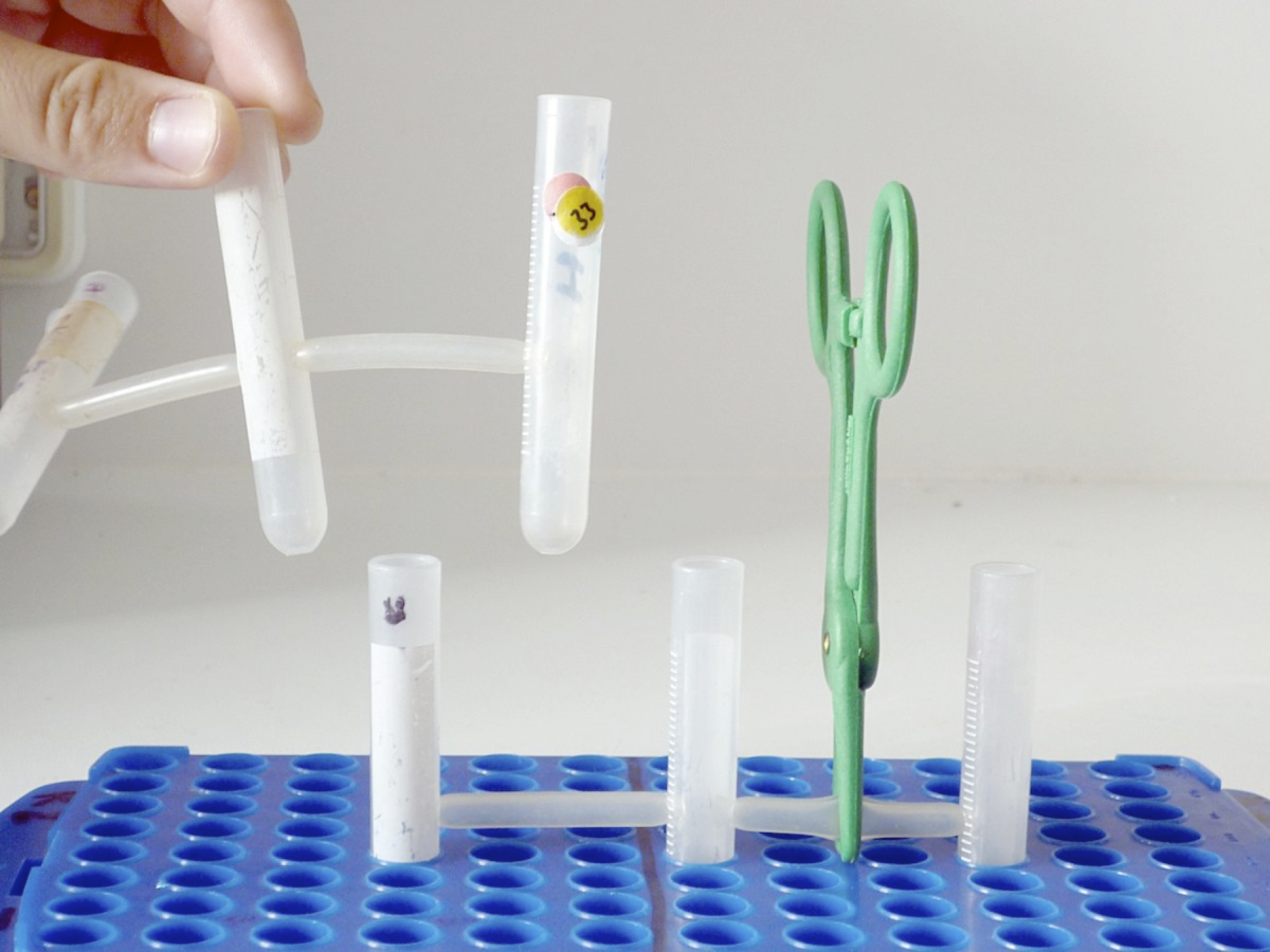

Supplement: Figure S1 — (TIFF) [file pone.0048436.s001.tiff]

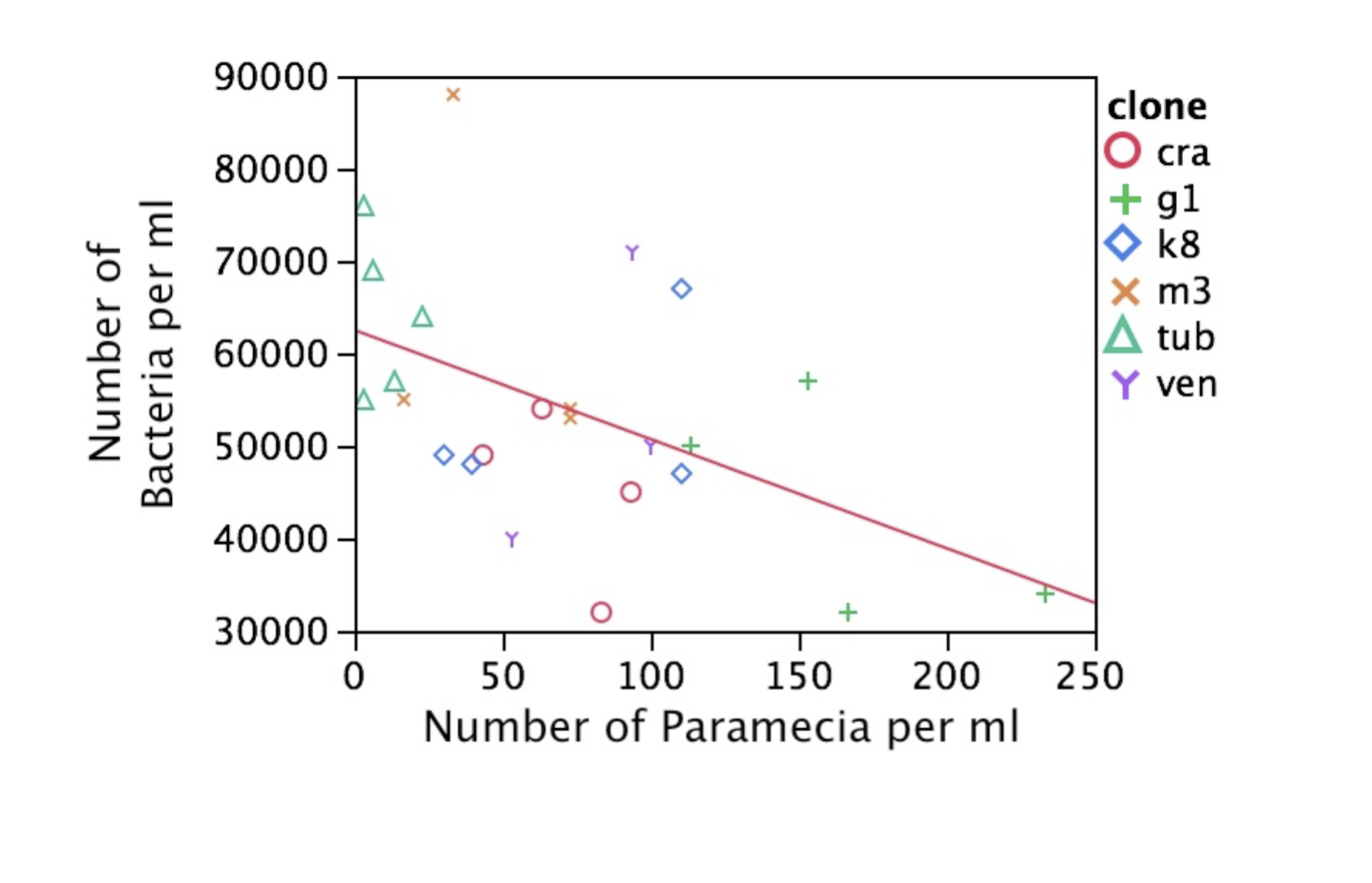

Supplement: Figure S2 — (TIFF) [file pone.0048436.s002.tiff]
