# Supplementary material for: Quorum Sensing and Density-Dependent Dispersal in an Aquatic Model System
Source: PLoS One. 2012 Nov 7;7(11):e48436. doi: 10.1371/journal.pone.0048436 (PMC3492347; doi:10.1371/journal.pone.0048436)
Supplement: Text S1 — (DOC) [file pone.0048436.s003.doc]

Text S1:

When we centrifuge paramecia to manipulate their density, it is possible that the bacteria, which serve as food, are also concentrated. In this case, treatments with higher paramecium densities might also contain greater numbers of bacteria, possibly inciting the cells to stay where food is rich. The above line of reasoning predicts low dispersal in bacteria-rich microcosms. It is parsimonious to assume that the same factor influenced dispersal in the experiment 2 and in our experiments with populations at natural densities (experiment 1 and experiment in Fellous et al. 2010). Then, these types of populations should also exhibit the association between high bacterial density and low dispersal. We know that unaltered populations with low dispersal are the ones with high ciliate density, according to the above hypothesis they should also contain high concentrations of bacteria. We tested for the presence of this relationship using 24 *P. caudatum* populations of 6 different clones, counting bacterial cells with standard bacteriological technics (i.e. plating on agar medium). We found the opposite of the predicted pattern: high ciliate densities associated to low bacterial numbers (Fig S2). This result, in accordance with traditional predator-prey dynamics, indicates that food-bacteria concentration does not explain the negative dependent dispersal of *P. caudatum*.
